# Peaks2Image: Reconstructing fMRI maps from stereotactic coordinates to enhance cognitive meta-analysis

**DOI:** 10.1162/IMAG.a.1367

**Published:** 2026-09-18

**Authors:** Raphaël Meudec, Jérôme Dockès, Fateme Ghayem, Demian Wassermann, Bertrand Thirion

**Affiliations:** Université Paris-Saclay, Inria, CEA, Palaiseau, France

**Keywords:** fMRI, coordinate-based meta-analysis, set transformer, image reconstruction, DiFuMo, decoding

## Abstract

To integrate novel findings and reach a consensus on the neural correlates of cognitive functions, human neuroscientists have relied heavily on the literature to formulate, validate, or refine hypotheses. Since thousands of publications have reported the coordinates of regions identified in brain mapping, the community has largely performed meta-analyses based on these coordinates. A known problem with coordinate-based meta-analyses is that they rely on limited information from each publication, so terms appearing in few publications are not reliably mapped. Recently, the use of public images has stimulated meta-analysis, which has become possible due to the creation of large image repositories. NeuroVault is a prominent example of such a repository. These repositories allow researchers to compare images across publications, leading to image-based meta-analyses. However, image-based meta-analysis faces a major obstacle: the scarcity and inconsistency of image annotation and metadata. In this paper, we propose *Peaks2Image*, a neural network approach that reconstructs continuous spatial representations of brain activity from peak activation tables. Peaks2Image provides dense brain reconstructions that can be combined with extensive annotations from the neuroscience literature. We use these annotations to train a decoder with textual features as labels. This results in a much broader set of decoded terms than current image-based studies. We validated the decoder using 47,000 annotated images and successfully identified 91 out of 108 terms, with a model that was uniquely trained using the neuroscience literature.

## 1 Introduction

In recent decades, significant neuroscientific research has focused on mapping human cognition and dividing it into processes within the brain. Applications range from gaining insight into what makes us tick to improving post-surgical recovery. Importantly, human brain mapping remains a consensus-based science: evidence for the involvement of one or more brain regions in a cognitive process is largely determined by the neuroscience community’s agreement [(Poldrack et al., 2017; Wager et al., 2007).

Different meta-analysis tools have emerged to infer agreement across the neuroscience community (Dockès et al., 2020; Menuet et al., 2022; Poldrack, 2011; Wager et al., 2007). These tools aggregate knowledge from the literature and extract agreement on how cognition is mapped onto the brain in a reproducible and quantifiable manner (Wager et al., 2007). Additionally, meta-analysis offers a partial solution to the pervasive issue of small sample sizes in neuroscience by providing priors that constrain data analysis and mitigate the risk of false detections. This is often implicitly implemented by masking strategies that build a binary mask of relevant regions from the literature, thereby reducing the space of hypotheses under investigation.

Interpreting results from neuroimaging studies presents challenges, especially regarding the specificity of the neural correlates of cognitive processes. When a cognitive task is studied in isolation, it is impossible to determine whether the observed brain activations are specific to the mental functions of interest or associated with a broader set of mental processes (Poldrack, 2011). One way to identify more specific associations between brain activity and mental function is *decoding*, or inferring the mental processes involved from a brain image of neural activity, especially when taking into account many alternative cognitive domains. To discriminate between a wide variety of cognitive states, a decoding model must identify brain regions that characterize each state rather than regions that are merely consistently activated (Varoquaux et al., 2018). For these associations to be specific, it is crucial that diverse cognitive states are decoded *jointly*. Due to the difficulty of formalizing the mental states associated with a brain image, high-quality labels are lacking for this supervised task. Performing large-scale decoding, sometimes called ”open-ended”, is, therefore, challenging. Thus, most decoding efforts only discriminate a limited set of cognitive concepts.

*Automated* meta-analysis methods (Laird et al., 2005) have been introduced to properly handle the growth of neuroscientific publications in recent years. NeuroSynth is a prominent example (Yarkoni et al., 2011). Handling neuroscientific concepts properly has emerged as a challenge for automated Coordinate-Based Meta-Analysis (CBMA), leading to the use of more complex textual features and models. *Neuroquery* (Dockès et al., 2020) broadened the spectrum of terms analyzed by mapping rare concepts to more common cognitive terms. *Text2Brain* (Ngo et al., 2021) leveraged language models (Beltagy et al., 2019) to capture semantic relationships between terms, enabling the encoding of any query. However, CBMA suffers from the drastic information reduction inherent to peak reporting (Salimi-Khorshidi et al., 2009).

Using dense images provides more information for meta-analysis. Thanks to the rise of large-scale databases of fMRI brain images such as NeuroVault (Gorgolewski et al., 2015) or OpenNeuro (Gorgolewski et al., 2017), decoding can be achieved across multiple studies (Mensch et al., 2017; Walters et al., 2022), making it robust with respect to study idiosyncrasies. However, the annotations associated with these images are often of low quality. Strategies that automatically improve labeling are necessary to properly benefit from the scale of the data (Poldrack & Yarkoni, 2016). The Neural Network on Dictionary (NNoD) (Menuet et al., 2022) framework leverages the Cognitive Atlas (Poldrack et al., 2011) to improve the labels for NeuroVault images, enabling the decoding of a large set of concepts. Overall, neuroscience meta-analyses have increasingly relied on data-driven approaches to learn more efficiently across large sets of data (Beam et al., 2021).

There have been few attempts at generative approaches to increase the amount of data (Zhuang et al., 2019), to generate or reconstruct fMR images (Cai et al., 2016; Manning et al., 2018), or apply self-supervised methods to neuroimaging signals (Thomas et al., 2022). However, there is still no systematic solution for reconstructing statistical brain maps from peaks, likely due to the daunting dimensionality of the data. The only approach we know of is the *Peaks2Maps* method, presented in Gorgolewski et al. (2019). Notably, we have not identified any attempts to integrate the rich textual content of neuroscientific publications with the dense spatial data of brain images.

This study presents *Peaks2Image*, a method for reconstructing dense brain images from peak coordinates contained in Coordinate-Based Meta-Analysis (CBMA) databases.^1^ *Peaks2Image* builds an enriched version of CBMA databases, enabling the decoding of dozens of cognitive terms from Image-Based Meta-Analysis (IBMA) databases without label supervision. Through this enriched database, we also created the first coordinate-based functional brain atlas, called the *Peaks2Image atlas*.

In this regard, we used peak coordinates, also known as stereotactic coordinates, which summarize the results of group studies in human neuroscience. We mapped these coordinates to dense activation images. Specifically, we used an unlabeled dataset of brain images to extract peaks and train *Peaks2Image* to infer images from which these peaks could be extracted. This process amounts to rebuilding full-brain activity landscapes from the maxima of these maps.

To evaluate Peaks2Image’s performance, we decoded cognitive terms from the largest available IBMA-compatible repository, NeuroVault (Gorgolewski et al., 2015). For each study, we extracted the reported cognitive processes, called labels or terms, by leveraging the term-frequency inverse document frequency of the study’s text. Validation is not trivial. It should provide quantitative information and predictive validity, that is, it should provide testable information on a set of fresh observations. For this, we turn to decoding (Cox & Savoy, 2003; Varoquaux & Poldrack, 2019). We used the decoding architecture from Neural Networks on Dictionaries (NNoD) (Menuet et al., 2022) trained on a neuroscience literature corpus. We evaluated the decoding performance against 108 terms used as NeuroVault annotations.

This approach leverages the rich descriptions of mental processes found in the literature and the high-quality neural activity data found in full-brain statistical maps. Our experiments demonstrate that Peaks2Image successfully decoded 91 out of 108 cognitive terms from 47,000 brain images from the NeuroVault database. These results suggest that Peaks2Image can decode a large set of cognitive terms using the CBMA and IBMA databases without any labeling supervision.

## 2 Methods

This section introduces *Peaks2Image*, our contribution to the field. Peaks2Image is a model that reconstructs statistical images from activation peaks. Our goal is to transform the sparse information from neuroscientific literature into dense spatial images. While Activation Likelihood Activation remains the most popular approach FOR CBMA (Yeung & Wong, 2025), Kernel Density Estimation (KDE) has been popularized by NeuroSynth, and both approaches are based on a light generation model based on Gaussian kernel. Here, we adopt a distinct approach by framing it as a learning problem. The learned model can build a dense spatial representation from any set of activation peaks, particularly those reported in the neuroscientific literature. We demonstrate the relevance of augmenting literature reports with such reconstructions by decoding cognitive processes without any labeled supervision.

First, we introduce the KDE representation in Section 2.1. Then, we describe the reconstruction task (Section 2.2) and the *Peaks2Image* model (Section 2.3). In Section 2.4, we describe the decoding experiments from the neuroscience literature used to study the decoding task. Finally, Section 2.5 presents the variants of the Peaks2Image model that we investigated. The Supplementary Material provides a detailed description of the data collection from the neuroscience literature.

In the following formulae, we represent scalars with lowercase characters, vectors as bold lowercase characters, and matrices with bold uppercase characters. For m∈ℕ , 〚m〛 will denote the {1,⋯,m} set.

### 2.1 Kernel density estimation

The typical procedure for peak extraction from task fMRI involves thresholding brain maps at a given level, for example, p<0.05 , FWER- or FDR-corrected, or p<0.001 uncorrected, and retaining only the large connected components. Then, local maxima are reported that are, for example, 8 or 12 mm apart among these supra-threshold regions. Thus, an image containing hundreds of thousands of voxels is reduced to a few coordinates—fewer than 100 for most studies. Recent meta-analysis models (Dockès et al., 2020; Laird et al., 2005; Ngo et al., 2021) often represent these few data points with a Kernel Density Estimation (KDE).

We compare the *Peaks2Image* model with recent KDE-based approaches. We use the *NeuroQuery* procedure to generate KDE representations from peaks. From a given set of peaks, we create a representation by placing a Gaussian kernel κ_σ_ at each peak position, with a full-width at half-maximum σ. Following previous work (Dockès et al., 2020), we set σ = 9* mm*. Formally, we consider the set of peaks $\text{A}\left(\text{y}\right)=\left\{{\text{a}}_{1},...,{\text{a}}_{l\left(\text{y}\right)}\right\}$ extracted from image y, where $\forall i\in 〚l\left(\text{y}\right)〛,{\text{a}}_{i}\;\in {\mathbb{R}}^{3}$. Given a fixed sampling grid $\text{G}=\left({\text{g}}_{1},...,{\text{g}}_{m}\right)$ representing the voxels, where $\forall j\in 〚m〛,{\text{g}}_{j}\;\in {\mathbb{R}}^{3}$, and the above-defined Gaussian kernel κ_σ_, we define the KDE representation ${\text{x}}_{\text{y}}$ that samples A(y) on the grid G:

$${\text{x}}_{\text{y}}\;=\left(x_{1},...,x_{m}\right),\;\text{where }\forall j\in 〚m〛:x_{j}\;=\sum_{i=1}^{l\left(\text{y}\right)}\kappa_{\sigma }\left({\text{a}}_{i},{\text{g}}_{j}\right).$$ (1)

This heuristic may not fully capture the co-occurrence of specific active locations, which is believed to be a hallmark of cognitive functions. The motivation for learning more sophisticated image reconstructions is to capture the information carried by the co-occurrence of locations and to obtain more informative representations of each publication.

### 2.2 Reconstructing brain activation images

The few data points available in stereotactic tables are derived from intricate activation patterns. Our goal is to reconstruct the statistical images that produced those data points. This reconstruction problem is an inverse problem involving the set of peaks and the image from which they were extracted. From a given set of *l* activation peaks $\text{A}\left(\text{y}\right)\in {\mathbb{R}}^{3\times l}$ extracted from image $\text{y}\in {\mathbb{R}}^{m}$ with *m* voxels, we learn the transformation $f_{\theta }:{\mathbb{R}}^{3\times l}\mapsto {\mathbb{R}}^{m}$ by minimizing the ${\text{L}}_{\text{2}}$ error over the original image:

$$f_{\theta }^{*}=\underset{\theta }{\text{arg min}}\;\;E_{\text{y}}{\left\|\;f_{\theta }\left(\text{A}\left(\text{y}\right)\right)-\text{y}\;\right\|}_{2}^{2}.$$ (2)

Since population-level images represent the average of individual data with variable shapes, high resolution is unnecessary. Therefore, we can perform a drastic reduction in the dimensions of the data, which is desirable given the limited sample size. We use the Dictionary of Functional Modes (DiFuMo) probabilistic atlas (Dadi et al., 2020), which efficiently reduces the dimension of the data from *m* = 50*K* voxels to *d* = 1,024 components. DiFuMo is a linear decomposition similar to principal components analysis, with additional constraints of non-negativity and sparsity on the atoms of the dictionary. It has been trained on thousands of resting-state and task fMRI scans and can, therefore, represent population-level information (Thomas et al., 2022). Using the DiFuMo atlas instead of the raw voxels helps us maintain a relatively low number of parameters.

Mapping voxels $\text{y}\in {\mathbb{R}}^{m}$ to coefficients $\alpha \in {\mathbb{R}}^{d}$ along DiFuMo dictionary $\text{D}\in {\mathbb{R}}^{m\times d}$ is simply done by linear regression, as shown in Eq. (3). All subsequent steps are performed in the *d*-dimensional DiFuMo space.

$$\alpha \left(\text{y}\right)=\underset{\alpha \in {\mathbb{R}}^{d}}{\text{arg }\min}{\left\|\text{y}-\text{D}\alpha \right\|}_{2}^{2}.$$ (3)

Therefore, Eq. (2) turns into learning the transformation $f_{\theta }:{\mathbb{R}}^{3\times l}\mapsto {\mathbb{R}}^{d}$ by minimization of the *L*_2_ error over the DiFuMo representation coefficients of the original images:

$$f_{\theta }^{*}=\underset{\theta }{\text{arg }\;\min}E_{\text{y}}{\left\|f_{\theta }\left(\text{A}\left(\text{y}\right)\right)-\alpha \left(\text{y}\right)\right\|}_{2}^{2}.$$ (4)

To solve the minimization problem in Eq. (4), we use a training set of statistical maps from NeuroVault, an online repository of fMRI maps. We extract peak coordinates from these maps. We standardize these images to eliminate the influence of maps with extremely high statistical values. Standardization also facilitates model convergence. Details on peak extraction are provided in the Supplementary Materials. This yields a training set of peaks and corresponding brain images that can be reconstructed.

The reconstructed images are obtained from the predicted DiFuMo coefficients via linear combinations of the dictionary components:

$$\hat{\text{y}}=\text{D}f_{\theta }^{*}\left(\text{A}\left(\text{y}\right)\right).$$ (5)

In the rest of the paper, the term *brain image* refers to its *d* = 1,024-dimensional DiFuMo representation coefficients α.

### 2.3 Peaks2Image

Peaks2Image uses a Set Transformer architecture (Lee et al., 2019; Zaheer et al., 2017) to solve Eq. (4). The transformation from a given set of peaks A(y) into the DiFuMo representations α(y) of an image can be decomposed into an encoder $\phi :{\mathbb{R}}^{3}\to {\mathbb{R}}^{h}$ applied to each element of the set followed by a decoder combining a pooling function pool and a row-wise feedforward layer *ρ* such that $\rho \circ \text{pool}:{\mathbb{R}}^{h}\to {\mathbb{R}}^{d}$, where *h* is the size of the hidden representations.

$$\alpha \left(\text{y}\right)=\rho \left(\text{pool}\left(\left\{\phi \left({\text{a}}_{i}\right),{\text{a}}_{i}\in \text{A}\left(\text{y}\right)\right\}\right)\right).$$ (6)

There are several reasons to use a set transformer architecture for image reconstruction based on peak location occurrences. The pooling function’s permutation-invariant property is highly relevant for peak locations because publications often do not report the statistical values associated with activation peaks. Therefore, no particular ordering of peaks can be inferred from the activation tables. Additionally, using sets of peaks as input eliminates the need for an intermediate representation. Thus, the Set Transformer model replaces the standard reconstruction obtained by applying a Gaussian kernel at peak locations, as described in Dockès et al. (2020), Laird et al. (2005), and Ngo et al. (2021). It also captures co-activation patterns induced by functional networks across the brain. Therefore, we compare Peaks2Image with KDE representations and with Peaks2Maps (Gorgolewski et al., 2019), a Pix2Pix (Isola et al., 2016) architecture trained on NeuroVault statistical images.

In practice, the Set Transformer accepts a fixed-size input. Therefore, we set the maximum number of peaks to be selected at 150. Since only a few publications report more than 150 peaks, we randomly trim the table for those publications. For publications reporting fewer than 150 coordinates, we pad the set with zeros to obtain the expected input size. We also pass a mask to the model to distinguish relevant peaks from padded ones. This mask ensures that the padded peaks are not considered in the pooling function.

### 2.4 Outputs: A functional brain atlas and a decoder trained using all available literature

Peaks2Image can reconstruct low-resolution brain maps from any set of peak coordinates. We use it on neuroscience publications to create such low-resolution brain maps with rich textual annotations, which reduces the technical gap between CBMA and IBMA. Using the NeuroQuery (Dockès et al., 2020) dataset and Pubget (Dockès et al., 2023), we gathered 19K neuroscience publications with stereotactic coordinates from PubMed Central. The tool parses the activation tables of these publications, totaling 400K peaks. We detail the data collection process further in the Supplementary Material. Note that the tables from each article were combined for reconstruction (meaning that the coordinates reported in the different tables were pulled together), as there is no simple way to leverage the association of the tables with the text. These maps can then be used to produce statistical summaries of the existing literature in the form of familiar brain maps. Therefore, we need to associate the reconstructions with descriptions of specific cognitive processes. Pubget extracts textual information from publications along the stereotactic coordinates. On top of this raw extraction, Pubget builds features such as the TF-IDF (Salton & Buckley, 1988) across a large vocabulary of 6,308 terms related to the field. The TF-IDF, which is also used in NeuroQuery and Text2Brain (Dockès et al., 2020; Yarkoni et al., 2011), reflects the relevance of a term to a study. It does so by giving more importance to terms that appear repeatedly in a few publications. We set a threshold at the 95th percentile of the TF-IDF to assign binary labels to publications, typically considering a few hundred positive samples per label. This ensures that the few publications most relevant to each term receive positive labels. The computed percentile may be 0 for some extremely rare terms. In this case, we only consider publications with strictly positive TF-IDF values. The result is a *functional brain atlas* in which each cognitive term receives a topography representing the meta-analytic reconstruction of that term.

To validate the reconstructions and their association with cognitive labels, we propose using a scheme that enforces external validity through out-of-sample generalization: decoding (Varoquaux & Poldrack, 2019). Thus, we decode statistical images from NeuroVault to identify the cognitive labels associated with each sample. The ability to decode cognitive concepts from the reconstructions ensures their validity and circumvents biases inherent to image reconstruction quality metrics. For example, the confounding effect of smoothness has been studied in Triantafyllou et al. (2006). Since we rely on information from the neuroscientific literature, we eliminate the constraint of having only a few labels associated with each brain image. We infer a much larger set of labels from the text of each publication. For the first time in cognitive neuroimaging, we perform decoding without label supervision by using only the peak activation tables in the most comprehensive public literature corpus. Using this dataset, we decode 108 cognitive processes from unseen NeuroVault images in a domain adaptation setting.

The Supplementary Material provides information on data collection and preparation, as well as training details.

Associating cognitive processes with brain images involves multi-label classification. The goal is to predict a set of labels $\text{L}={\left\{0,1\right\}}^{C}$, where *C* denotes the total number of classes, from the DiFuMo-space representation of an input image $\alpha \left(\text{y}\right)\in {\mathbb{R}}^{d}$.

We use a three-layer dense neural network that is trained using only neuroscience publications, without any supervision from NeuroVault samples or their labels. For model evaluation, we are limited by the available NeuroVault annotations. In the following experiments, we predict only *C* = 108 terms out of C = 6,308 considered in the TF-IDF vocabulary. However, note that Peaks2Image can be extended to a set of cognitive terms as large as the vocabulary used, with no additional labeling cost.

**Fig. 1.**
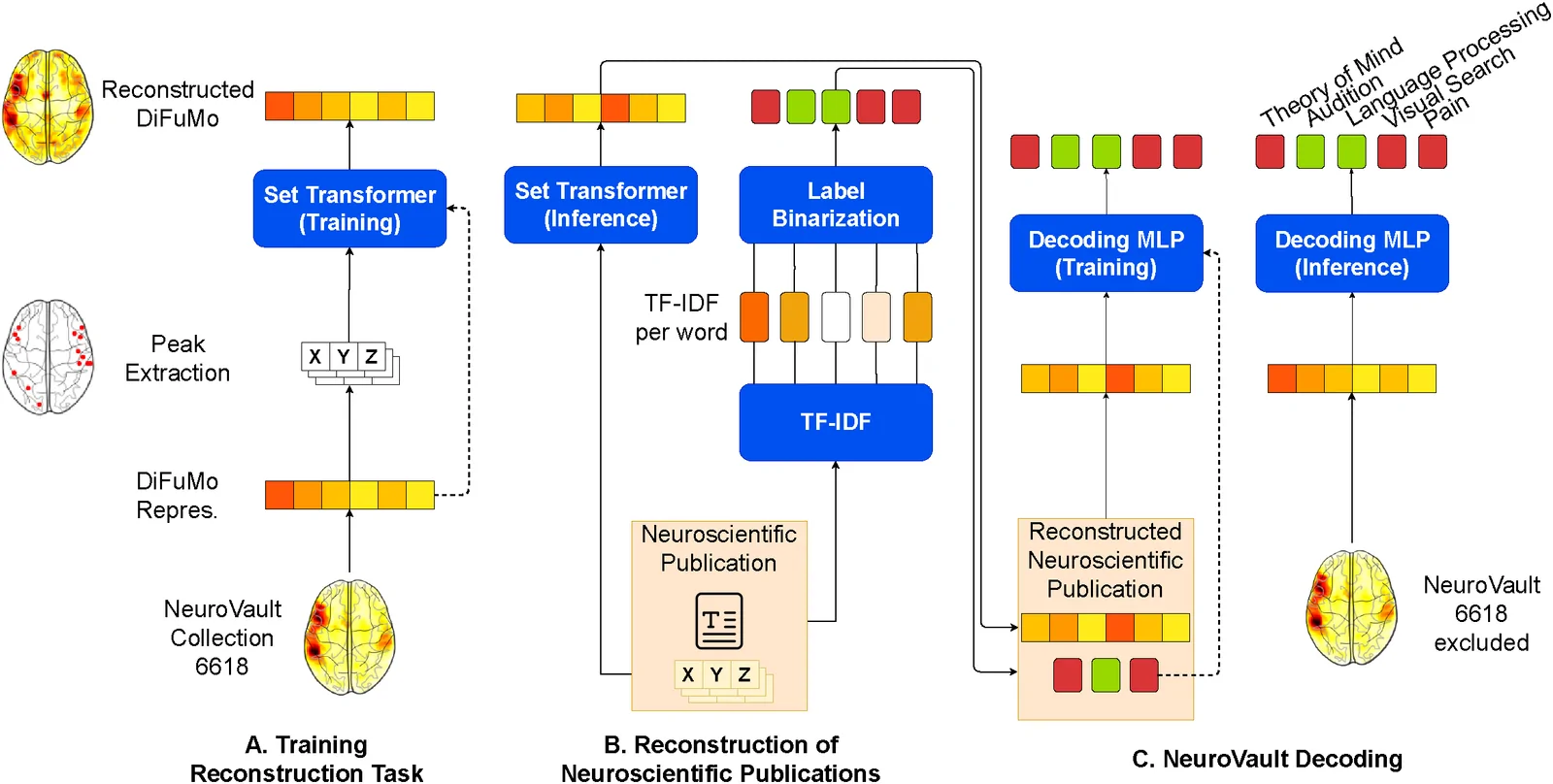
Reconstructing brain activation patterns from activation peak tables reported in the literature. (A) From a set of statistical images, we extract the peaks above a statistical threshold. We use a Set Transformer model to reconstruct the DiFuMo coefficients of the image after normalization. (B) Once the reconstruction model has been trained, it is applied to the coordinates from the peak activation tables in 19K scraped articles. This yields a brain map for each publication, which supports further image-based computations such as decoding. We extract the Term Frequency - Inverse Document Frequency (TF-IDF) values for each word from the article’s text and infer labels using a percentile-based threshold. (C) We then train an image decoder based solely on the literature. For validation, we test the decoder on the NeuroVault repository. We decode a broad set of cognitive processes benefiting from the large vocabulary without any label supervision.

Figure 1 shows a summary of Peaks2Image. It consists of three parts: learning the reconstruction process from peaks (A), applying the reconstruction to neuroscientific publications (B), and decoding brain images with a model trained from those reconstructions in a domain adaptation setting (C).

### 2.5 Use of alternative latent representations provided by image autoencoders

Dimension reduction is a key component in tackling this reconstruction problem. We use the DiFuMo atlas to reduce the dimensionality of the problem, making it easier to capture co-activation patterns. We implemented other dimensionality reduction schemes for comparison. In particular, we consider the latent space of an autoencoder (AE) trained on NeuroVault data (Germani et al., 2023). Using the latent representation of this convolutional AE, we continue to use latent variables that translate to local patterns, similar to DiFuMo loadings. This enables the AE-latent version of our model to compete with the DiFuMo version in terms of both Dice metrics (Dice, 1945) and AUC for the downstream multilabel classification task.

This alternative choice affects the Peaks2Image model in two different ways. First, the model no longer outputs a DiFuMo representation of an image but rather the latent representation of the autoencoder. The encoder is built from a series of convolutional and max-pooling layers that transform the original brain volume into a compressed representation of 3 x 4 x 3 voxels across 512 channels. This increases the dimensionality of the Peaks2Image output from the 1,024 DiFuMo components to an 18K latent space. Second, a necessary post-processing step is added to obtain images from the predicted values: applying the AE-based decoder. However, this does not drastically affect the model’s computational efficiency, as reconstructing images from DiFuMo components also requires postprocessing to obtain the reconstructed brain image.

We process statistical images from NeuroVault using the procedure described in the original paper (Germani et al., 2023). This yields a dataset that differs slightly from the task version of Peaks2Image. We replace the DiFuMo representations of the images with their latent representations in the autoencoder space. We flatten these latent representations to fit the set transformer for this output. Postprocessing involves reshaping the predictions to the original latent dimensions before applying the decoder to produce the final image prediction.

In all decoding experiments, we use the AUC, as the chance level is normalized to 0.5, even for highly imbalanced data. We performed 20 runs to obtain confidence intervals for each cognitive task, rotating the training and validation sets from the neuroscientific literature. Evaluations are carried out in voxel space on all NeuroVault statistical images excluding IBC, which is used in the reconstruction training.

## 3 Results

The experimental results are presented as follows: In Section 3.1 we quantitatively and qualitatively compare the reconstruction results obtained by Peaks2Image with those obtained by alternative techniques. Section 3.2 presents the results of the large-scale decoding experiment based on the neuroscience literature. In Section 3.3 we contextualize these results by comparing them with alternative techniques. We then perform two ablation experiments to better assess the methodological contribution: In Section 3.4, we examine the impact of the dimension reduction inherent in Peaks2image. We also compare the method with an alternative reconstruction based on ICA decompositions of resting-state data in Section 3.5.

### 3.1 From peaks to dense spatial images

Before applying this reconstruction model to the neuroscientific literature, we measure the quality of the reconstructions produced on NeuroVault images. We use the Dice coefficient (Dice, 1945) (as in Text2Brain (Ngo et al., 2021)) to evaluate the reconstructions produced by Peaks2Image on all NeuroVault statistical images, which represent a testing set of around 47K images. We excluded collection 6,618, which was used to train the Peaks2Image model. Given a threshold *t*, the parametric Dice coefficient *D_t_* measures how well voxels in $\hat{\text{y}}$ reconstructed with a value greater than *t* coincide with the voxels in the original image y. This eliminates correlation obtained in noise-only regions, and focuses on the most activated regions:

$$D_{t}\;=\frac{2*\sum 1_{\text{y}>t}1_{\hat{\text{y}}>t}}{\sum 1_{\text{y}>t}+\sum 1_{\hat{\text{y}}>t}}.$$ (7)

**Fig. 2.**
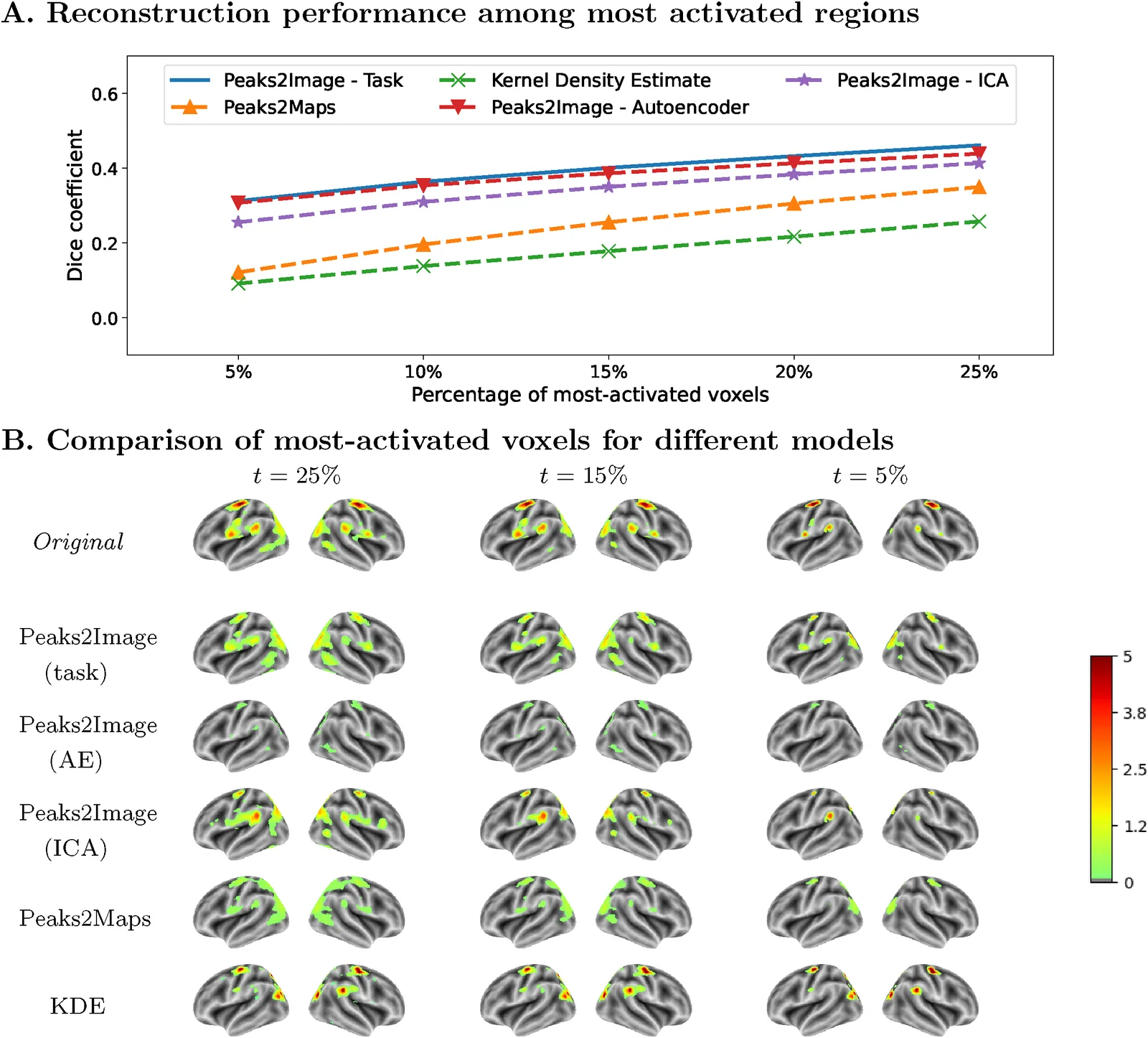
Reconstruction performance of NeuroVault statistical images: we reconstruct brain images taken from Neurovault from their peak coordinates. (A) We compute the Dice coefficient at different thresholds *t* (see Eq. (7)). Dice coefficients measure how accurately the most activated regions are identified in the reconstruction process. The three variants of Peaks2Image outperform the other representation methods, meaning that Peaks2Image reconstructions better capture the activation patterns of the most active regions. (B) We threshold the brain volume at different top *t*% thresholds and plot the projection of the thresholded maps on surface for different representations (from top to bottom, the NeuroVault original statistical image, Peaks2Image [task, autoencoder, ICA], Peaks2Maps, KDE). Peaks2Image provides a more truthful reconstruction than the other methods, capturing co-occurrences patterns that are not visible with other representations.

Although the model was trained using mean-squared error in DiFuMo space, we find that the Dice coefficient is a more interpretable measure of reconstruction quality. We set the threshold at different percentile values of the original image to validate the model’s ability to recover the most active regions. We present the reconstruction performance of each model in Figure 2. Some examples of reconstruction with Peaks2Image are given in Supplementary Figure S1.

As shown in Figure 2, Peaks2Image outperforms all other representations of statistical images. The KDE representation appears to be suboptimal because the resulting dice values are low at all thresholds. Simply positioning activation at the peak location is insufficient to capture the occurrence patterns among the most activated regions. The Gaussian prior in the KDE representation leads to either over-smoothed or over-activated regions.

We also consider Peaks2Maps (Gorgolewski et al., 2019), which uses a UNet architecture to reconstruct a brain image from the KDE representations. Peaks2Image produces regions that are more similar to the original image and misses fewer activations in regions with no reported peaks. This leads to larger Dice metrics. However, Peaks2Maps tends to produce reconstructions with less contrast (see Fig. 2), as if the convolutions were blurring highly activated voxels. Using DiFuMo representations instead of raw voxels helps accurately produce highly activated regions and capture co-activation patterns. To complement this result, we also show in Supplementary Figure S2 a per-sample measure of reconstruction accuracy, which confirms that Peaks2Image outperforms kernel-based reconstruction in a large proportion of the samples.

**Fig. 3.**
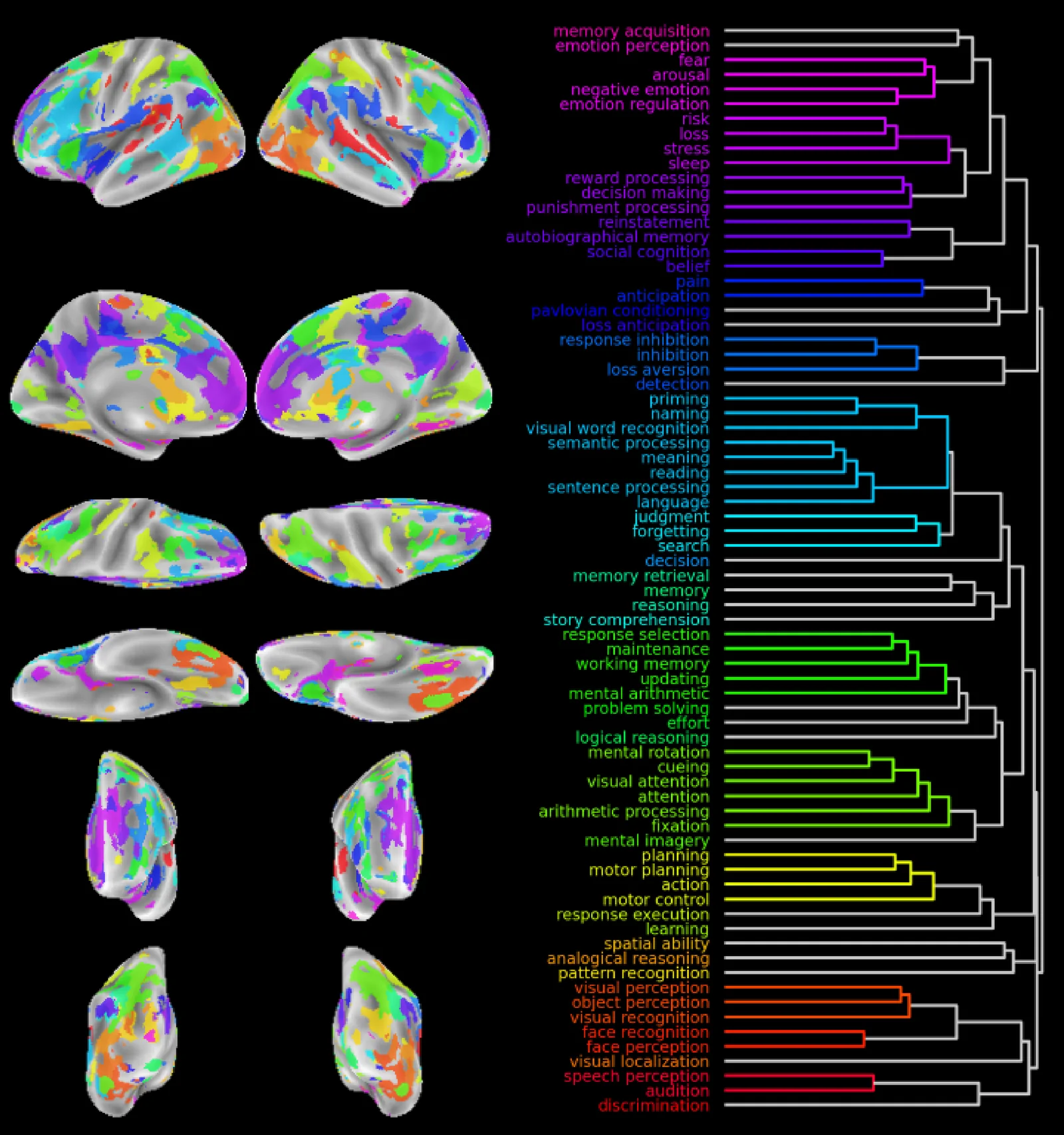
A summary functional brain atlas derived from the literature. (Left) The maps of the atlas are plotted after being thresholded to the upper 99th percentile. The color scheme is adjusted so that maps with similar content are the same color. (Right) Clustering of concepts according to the similarity of the reconstructed map. This representation is useful for summarizing the main consensus of the literature on brain topography. It also outlines cognitive concepts that activate similar brain areas. It also shows that some brain regions have not yet been assigned a clear functional role in the literature.

We use the reconstructions to create a task-specific brain atlas using contrast images obtained for each term in the TF-IDF vocabulary. For a given term, we create a contrast map using a two-sample t-test comparing the reconstructed images of the publications with a positive TF-IDF scores with the rest of the corpus. Unlike NeuroSynth or NeuroQuery contrast maps, Peaks2Image can reconstruct negative activations. We show the atlas projected onto the surface in Supplementary Figure S3. We also provide a synthetic representation of the set of components as a functional brain atlas shown in Figure 3. To simplify, we retain the upper percentile of each activation map and cluster the components according to their spatial similarity using a hierarchical clustering algorithm with average linkage. Consequently, the clustering algorithm groups contrast with a similar topography and plot them together. Interestingly, this clustering leads to the following chains of concepts: emotion, value, self-representation, inhibition, language, reasoning, executive function, mathematics, visuo-spatial functions, motor, vision, and audition. This is compatible with the gradient view that emphasizes the difference between primary and perceptual systems (Margulies et al., 2016), and more generally, the distinction between primary and associative, as well as higher-level, functions. Another observation is that not all brain regions are covered equally by the literature, either because they are difficult to map with functional neuroimaging (e.g., anterior temporal regions) or because their function is difficult to capture.

### 3.2 Brain decoding from the neuroscientific literature

We evaluate the relevance of reconstructions by using a model trained only on reconstructed neuroscientific publications to identify cognitive labels associated with NeuroVault images. This model has never encountered actual brain images; it has only seen texts from the literature. In this domain adaptation setting, we do not rely on NeuroVault annotations for training, which lifts the constraint of annotation availability for each brain image.

Identifying cognitive labels associated with brain images is a multi-label classification task that we evaluate by computing the ROC AUC (Dice, 1945) over each label. We evaluate the decoding of all NeuroVault images not used to train the reconstruction model. Similarly to NNoD (Menuet et al., 2022), we apply label enrichment to the original image labels. This process uses regular expressions to map different terminologies for the same concept, as well as a hierarchy-based inference of labels based on the Cognitive Atlas ontology. For a given term, we also assign parent terms from the ontology. These procedures are described in detail in the Supplementary Material of the NNOD paper.^2^

**Fig. 4.**
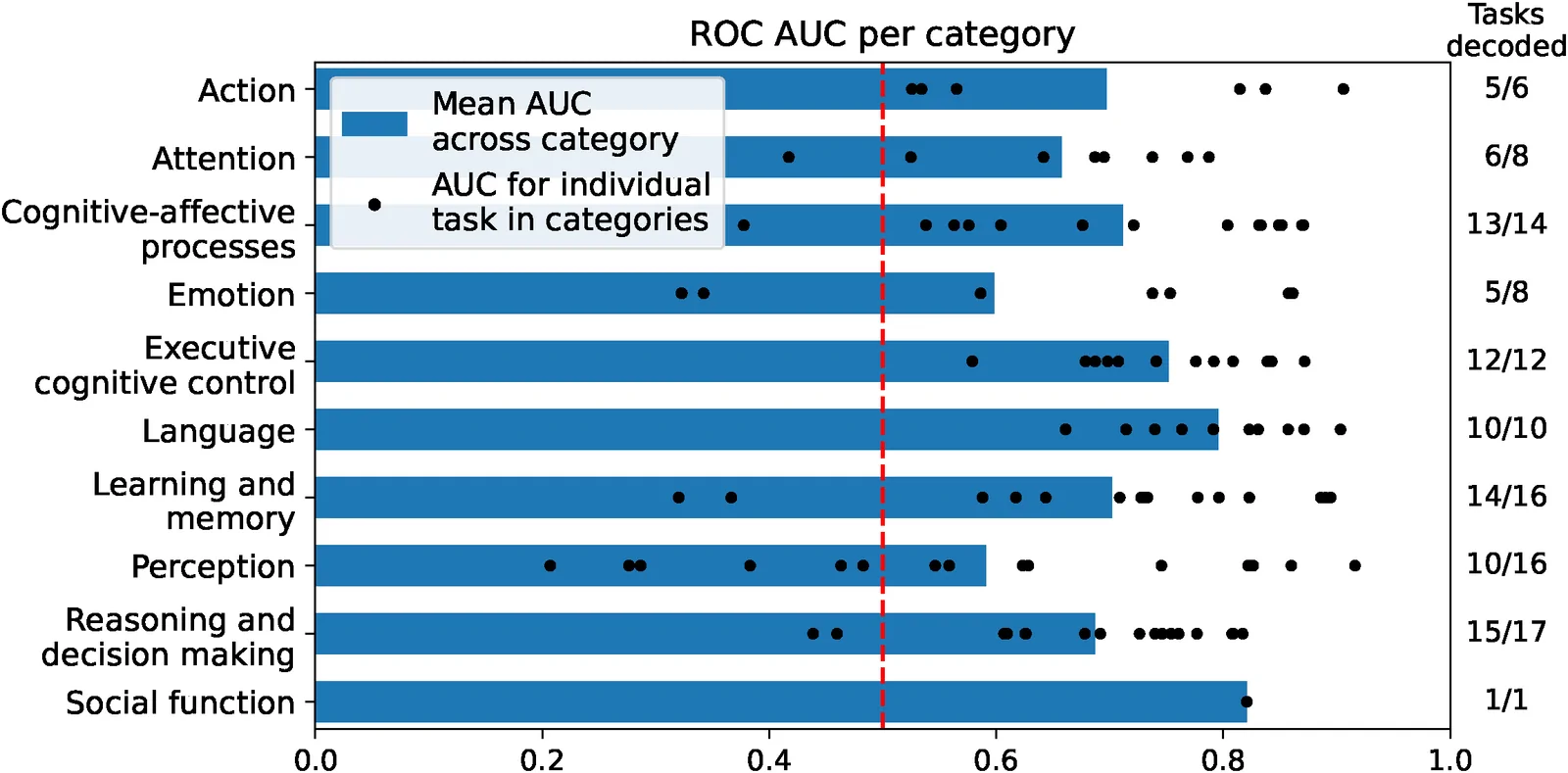
Decoding performance on NeuroVault: We evaluate the relevance of the reconstructions by identifying the cognitive labels associated with the NeuroVault images. Peaks2Image provides reconstructions for the neuroscientific literature, which we use to train a decoder by associating labels with TF-IDF representations of the text. The resulting decoder performs better than chance on 91 out of 108 cognitive processes over 47K images (AUC > 0.5 over at least 95% of the runs). Evaluation is performed on all NeuroVault except collection 6,618. We excluded terms with fewer than 20 positive examples to ensure reliable results.

We decoded 91 cognitive terms out of 108 among 47K NeuroVault images, without any NeuroVault label supervision. We summarize the decoding performance by aggregating over Cognitive Atlas categories in Figure 4. The affiliation of each cognitive term to Cognitive Atlas categories is listed in Supplementary Table S1, and the per-term AUC values are given in Supplementary Table S2. Peaks2Image performs well across all main concept families of the Cognitive Atlas.

**Fig. 5.**
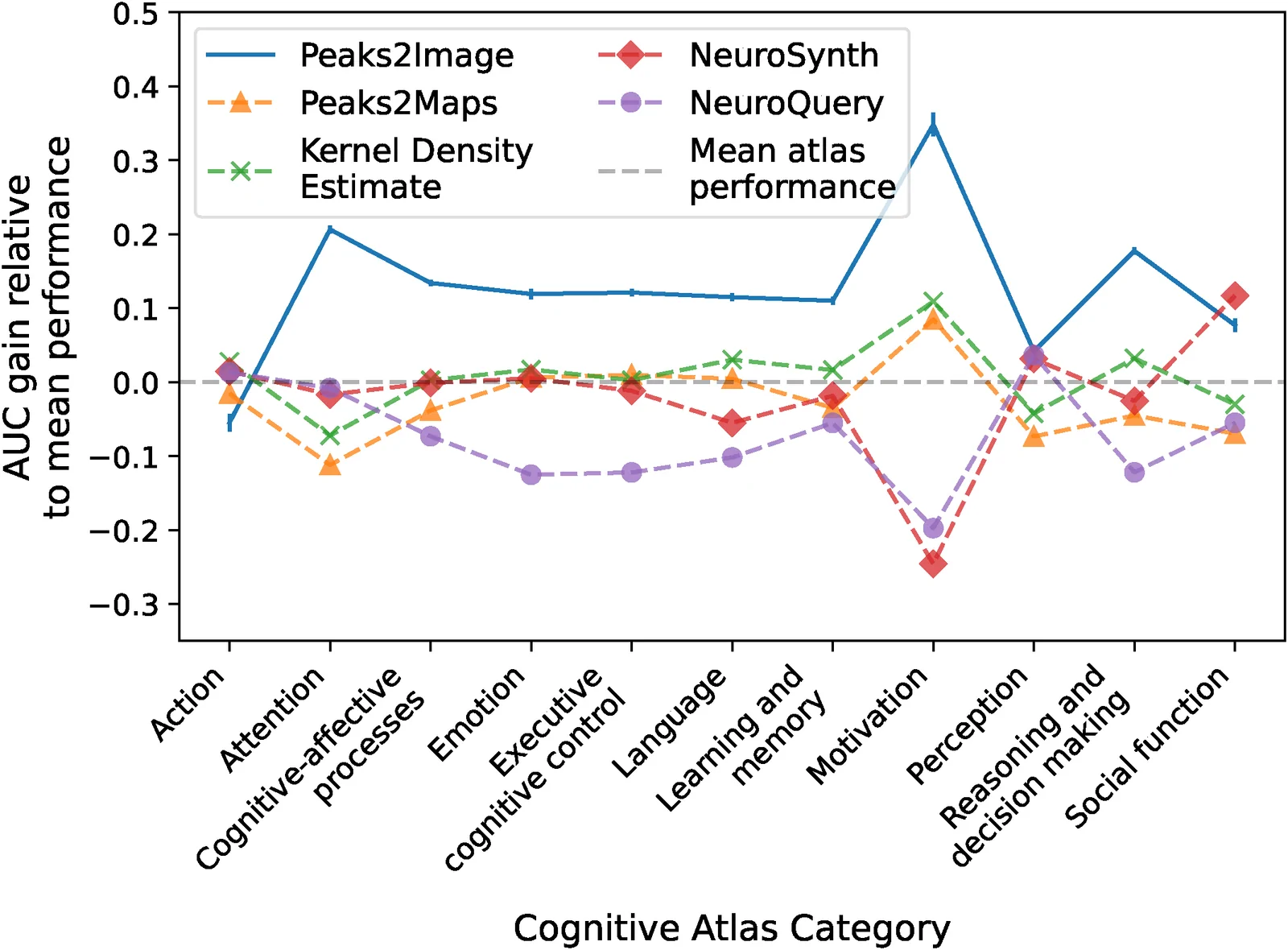
Relative AUC performance across tasks compared with mean performance of all reconstruction methods: We decode NeuroVault terms using each representation method as the training set. Then, we compute the AUC gain for each word relative to the mean performance. Then, we plot the aggregation of those gains across Cognitive Atlas categories. Peaks2Image largely outperforms other approaches in most categories.

The reconstruction process plays a significant role in decoding performance. Thus, we compared the Peaks2Image reconstructions and the KDE representations used in the literature, as well as the Peaks2Maps, by assessing their performance in the decoding task. Peaks2Image outperforms all of these models in the decoding task (see Fig. 5). We note that, although Peaks2Maps relies on a learning process for reconstruction, it performs worse than the KDE for decoding. We believe this is due to the lack of specificity in the reconstructed brain images. Peaks2Maps poorly reconstructs high-intensity voxels (see Fig. 2), which probably inhibits the decoder from capturing the effect of specific voxels on cognitive processes.

### 3.3 Comparison with CBMA methods

We evaluate the strength of our reconstruction process by directly comparing it with coordinate-based methods, such as NeuroSynth and NeuroQuery.

Using Pubget, we fit the NeuroSynth and NeuroQuery models on the same corpus as Peaks2Image. We compare the Pubget version of NeuroSynth with the original NeuroSynth.^3^ In the Appendix, we demonstrate that both methods achieve comparable decoding results for terms from the original vocabulary. Using NeuroSynth on the same corpus as Peaks2Image allows us to compare the full vocabulary, not just the cognitive terms handled by the standard interface. As with Peaks2Image, we use the decoding capabilities of the NeuroSynth model to predict terms shared with the NeuroVault vocabulary. We use the chi-squared test statistics from NeuroSynth to associate labels with each statistical image, leveraging the correlation.

Since Pubget is developed by the NeuroQuery authors, we assume equivalence between the initial and fitted models. As with NeuroSynth, we generate a statistical image for each term using the NeuroQuery model. Then, we compute the correlations of NeuroVault images with the reference maps.

As shown in Figure 5, Peaks2Image outperforms both NeuroSynth and NeuroQuery in most Cognitive Atlas categories. The NeuroSynth model competes with Peaks2Image with respect to well-established terms such as “language” or “memory”, but it performs poorly with more specific terms.

### 3.4 Dimension reduction benefits to decoding accuracy

Dimensionality reduction is a key component in tackling this reconstruction problem. Switching from DiFuMo loadings to autoencoder representations yields slightly different samples. We noted that it tends to reconstruct sharper patterns, but capturing co-activation patterns poses a greater challenge. We attribute this difficulty to the decoder’s depth; it comprises only four deconvolution layers. This probably limits its ability to reconstruct co-activation patterns distributed across the brain. Using the AE-latent version of our model has little impact on performance in the reconstruction (see Fig. 2) and decoding tasks. We find that the DiFuMo loadings are a simpler, more interpretable way to reconstruct images.

### 3.5 Training Peaks2Image with resting-state instead of task-fMRI data

Task-fMRI data are expensive to collect and annotate, whereas a large amount of resting-state data is easily accessible. We investigate using the abundant resting-state data to learn the reconstruction process. We evaluated the ICA-trained model on the same NeuroVault dataset (Gorgolewski et al., 2015), which required an additional adaptation to account for the dataset shift. As shown in Supplementary Figure S4, NeuroVault maps yield a more accurate reconstruction than resting-state ICA maps.

## 4 Discussion

This paper tests the hypothesis that generative AI approaches can solve the problems of scarce and inconsistent annotations and metadata in brain image databases. To test this hypothesis, we introduce Peaks2Image, a model that reconstructs brain images from peak activity coordinates. The concept of *reconstruction* is better understood in the context of modern AI, where it has been achieved using latent models, such as diffusion and generative models, in which the final data reconstruction is performed through an autoencoder. In this case, the latent representation is the Difumo representation, and the Difumo reconstruction plays the formal role of the autoencoder.

We note that the ability to reconstruct images from a set of coordinates is useful. For example, it could be used to compress neuroimaging activation data, sample brain images, or verify whether a given table contains sufficient information to identify the underlying pattern of activity. Peaks2Image bridges the gap between neuroscientific literature and brain image databases. It uses coordinates reported in publications to reconstruct dense, low-resolution images. Peaks2Image builds a database of dense spatial images based on publication content. This improves the spatial information in coordinate-based analyses and creates an image-based database with rich textual content. This database is the foundation for the first coordinate-based atlas of brain images derived from neuroscientific literature. Contrast maps can capture negative effects and probe the independence of certain brain regions in relation to specific cognitive tasks.

Peaks2Image builds dense spatial representations for neuroscientific publications. It improves upon the KDE that has been used in all CBMA so far, as well as in NeuroSynth and NeuroQuery. We note that such an improvement is hard to measure. The least controversial criterion that we could identify is the ability to decode terms associated with brain maps. This is why we ended up assessing the generalization of the topographic models built from the literature to decode an annotated image dataset. As shown in Figure 2, Peaks2Image reconstructions are more similar to brain images than KDE representations and better represent the most activated voxel patterns. Additionally, we measure the suboptimality of the KDE, which struggles to pinpoint the activation patterns commonly found in functional brain imaging. Our model, associated with TF-IDF features extracted from the publications, enables a more accurate and richer representation of each publication, further improving the description of each brain image. As shown in Figure 5, Peaks2Image outperforms NeuroSynth and NeuroQuery across most Cognitive Atlas categories, while expanding the number of cognitive processes that can be decoded. One might wonder whether poorly decoded terms would have well-decoded hypernyms. This appeared to be the case in some instances, such as when “mental arithmetic” received an AUC of 0.45 and “arithmetic processing” received an AUC of 0.79. However, this was not systematically the case. Compared with image-based models such as *NNoD* (Menuet et al., 2022), it enables the decoding of a broader range of cognitive processes by leveraging the textual information of neuroscientific publications and reconstructions, all while maintaining a good decoding performance. Most importantly, the decoding performance is reached *without additional labeling effort* on activation images. Such an effort is time consuming and error prone; yet, the level of accuracy raised in this work is not high enough to replace the community effort that consists in curating annotations associated with public data. This will be a necessary step in order to train and to validate potential future automated labeling approaches. This represents the literature’s first successful automatic decoding of 91 terms, among 108 that could be tested. To the best of our knowledge, this is twice the number reached in previous works in the field (Menuet et al., 2022). This limited evaluation reflects the scope of our test set. Although Peaks2Image can be extended to any term in the vocabulary, our evaluation is limited by the availability of labels on NeuroVault images. As a side note, the vast majority of maps used from NeuroVault are single-subject maps (such maps represent 88% of NeuroVault content). Having a larger proportion of group-level maps may make our test set less idiosyncratic to individual configurations and increase accuracy.

We use Peaks2Image to train on task-fMRI brain images, which are costly to collect and annotate. We investigate the impact of training on abundant resting-state data. Although resting-state data lack cognitive labels, it displays a rich repertoire of topographical maps that we extract through independent component analysis (ICA) (Smith et al., 2013). Our model can be trained using any set of peaks and their corresponding ground images. We extract components from randomized ICA runs on HCP resting-state data. We obtained a model for statistical image reconstruction that nearly matched the performance of the task-supervised version in terms of Dice score. Notably, we obtained a reasonably accurate brain map decoder that was never trained on actual task maps! The model achieves good overall correlations. However, reconstructions from resting-state data still have slightly lower decoding capabilities. This suggests that brain maps obtained through ICA components may lack the specific patterns necessary to distinguish cognitive processes properly, as discussed in Demirtaş et al. (2019) and Tuominen et al. (2023). This may be more attributable to the input data than to the ICA methodology. This gap could be bridged in the future, however.

Image compression is a key architectural element that allows Peaks2Image to capture patterns more effectively for reconstruction. We demonstrate that DiFuMo exhibits similar behavior to other compression strategies, particularly the latent representation of an autoencoder trained on NeuroVault images (Germani et al., 2023). Our findings confirm that this domain-specific dimension reduction is well suited to properly reconstructing brain images, as we observe similar reconstruction and decoding results. However, DiFuMo has several advantages over the autoencoder. For example, it captures co-activation patterns more effectively, as shown in Figure 2. The DiFuMo atlas representation is built around functional components best suited for characterizing co-occurring activation patterns. Additionally, the DiFuMo atlas is more easily interpretable than the autoencoder latent space, which uses several convolutional layers for image reconstruction. Nevertheless, we emphasize that we used DiFuMo for convenience in this work, but we would welcome any other atlas or parcellation with the same expressive power. We also note that many recent works on foundation brain model rely on parcellation- or region-based approaches, such as Careil et al. (2025), Caro et al. (2024), Menuet et al. (2022), and Thomas et al. (2022). Interestingly, none of these papers strongly argues for distinguishing between dense maps and the low-dimensional parametrization of such maps. To date, “foundation models” remain low resolution. In the future, one can expect higher-resolution models to perform better.

An important limitation of the presented framework is that it relies on a crude model of texts—a bag-of-words—to represent the semantics thereof. First, it hinders comparisons by limiting them to exact matches between vocabulary and tags from NeuroVault. Although Peaks2Image could be extended to include any word in the neuroscientific literature, the evaluation was limited to a small set of concepts due to the lack of extensively annotated brain images. Exploring more powerful language models as in Text2Brain (Ngo et al., 2021) is a future direction that could better exploit the continuous brain images generated for the neuroscientific publications. This approach should facilitate understanding the semantics of queries rather than reducing the description of an image to just a few terms. However, poor-quality NeuroVault annotations would still limit our ability to evaluate these models. Second, it does not include the context in which each word is used. Applying binary labeling based on TF-IDF values is most likely a crude approximation of each word’s semantic content. We believe that more advanced language models (Touvron et al., 2023; Wu et al., 2023) could better infer main topics of publications (see e.g., Ghayem et al., 2026). In short, Peaks2Image is a novel and powerful bridge between different representations of information about cognitive functions and brain activation demonstrated by this study, which should be further developed and validated.

Peaks2Image paves the way for improved generation of large sets of images for neuroscientific publications. It shows how to overcome limitations caused by poor image annotations using a domain adaptation approach. In particular, neuroscientific publications are a challenging application for generative approaches (Pinaya et al., 2022) and recent text-to-image representation learning methods (Radford et al., 2021). The Peaks2Image approach is well suited for enriching the cognitive labels of Neurosynth for further large-scale, image-based meta-analyses, as well as for providing an additional, potentially richer way of conducting reverse inference on brain maps such as Neurosynth.

## Supplementary Material

Supplementary Material

## Data Availability

The trained model is publicly available on Github,^4^ among the code to reproduce the experiments. We also include pre-computed DiFuMo representations of NeuroVault images and fetched publications from pubget. The code is released under the MIT license.

## Footnotes

1 It should be noted that positions reported in tables may also represent deactivations that reach a certain statistical significance. As is customary in CBMAs, we assume that these cases are in the minority and consistently refer to *peak* positions.

2
68870-supplement-Supplementary_Results_and_Discussion.docx

3 Implementation from https://www.github.com/neurosynth/neurosynth.
