# Supplementary material for: Peaks2Image: Reconstructing fMRI maps from stereotactic coordinates to enhance cognitive meta-analysis
Source: Imaging Neurosci (Camb). 2026 Sep 18;4:IMAG.a.1367. doi: 10.1162/IMAG.a.1367 (PMC13591505; doi:10.1162/IMAG.a.1367)
Supplement: Supplementary file 1 [file IMAG.a.1367_supp.pdf]

Suuplementary material to Peaks2Image: reconstructing fMRI maps from stereotactic coordinates to enhance cognitive meta-analysis

Supporting information

0.1 Neuroscientific literature

0.1.1 Data collection

We leverage Pubget[1] to fetch the neuroscientific literature, exploiting the Pubmed Central API. We fetch publications from an extensive list of journals[2], resulting in 400K neuroscientific publications. While this seems like a large number, only a small fraction of those publications contain stereotactic coordinates. We identify those and extract a subset of 19K publications from which we obtain both the text and the activation tables. The tool helps us parse the activation tables of those publications, totalling 400K peaks. It also transforms each study text into Term Frequency - Inverse Document Frequency (TF-IDF) features across a vocabulary of 6308 terms.

0.1.2 NeuroSynth and NeuroQuery models

Pubget also facilitates the preprocessing of the publications by providing directly the TF-IDF representation of the text. It facilitates the fit of NeuroSynth and NeuroQuery on the set of downloaded publications.

We use those versions to act as our baselines. We make sure that this newly fitted NeuroSynth version matches the original performance[3] on shared terms.

NeuroSynth computes statistical maps from publications by performing a Chi-squared test on the co-occrrence of terms and locations reported across publications. They perform the decoding through correlation of a given image to those Chi-squared maps. We observe similar decoding results between our fitted version through Pubget and the original NeuroSynth one, with a slight improvement of the updated version (AUC of 0.663 vs 0.655 for NeuroSynth). This difference in performance can be explained through two main factors. First, the set of publications used in both versions are different. The original NeuroSynth version uses a set of 11K publications, while our updated version uses over 19K publications. Hence, the Chi-squared maps obtained for each term differ slightly. Second, Pubget uses the full body of the publication, while NeuroSynth only uses the abstract. This expands the vocabulary reach of this updated NeuroSynth version, and allows decoding for a broader set of terms.

0.2 NeuroVault

0.2.1 Data collection

We assess the quality of the informations captured from the literature through the NeuroVault database. We exclude a subset of images according to a set of rules such as

[1] `https://www.github.com/neuroquery/pubget` - v0.0.8
[2] `https://github.com/neuroquery/pubget/blob/main/docs/example_queries/journal_list_fmri_vbm.txt`
[3] Implementation from `https://www.github.com/neurosynth/neurosynth`

having an incoherent range of values (indicating that an image does not contain Z or T statistics), duplicated images or images with missing metadata to keep 55K brain maps for our task. We split those maps into two different sets in a collection-wise manner. We take collection 6618, without labels (6K maps), to train the reconstruction task. We consider the remaining 47K brain maps from 2376 different collections to evaluate the performance on the decoding task.

For validation, we restrict the analysis to terms shared between the tags of NeuroVault images described in NNoD [Menuet et al.2022] and the vocabulary from the TF-IDF. Among the 240 tags from the NeuroVault test collections, 189 are present in the vocabulary. We focus on those 189 tags without adding any additional mappings between the left-out tags and words from the vocabulary. Additionally, we filter out terms that contain less than 20 positive examples out of the 47K images in the test set to prevent any non-significant effects, keeping 108 terms to decode. We list those rare terms below.

Filtered rare terms: acuity agency, agreeableness, anxiety, appetite, attentional blink, attentional state, attitude, auditory masking, balance, binocular rivalry, centration, cognitive effort, cognitive load, color perception, communication, competition, conceptual priming, consciousness, conversation, creative thinking, crossmodal, deductive reasoning, desire, distraction, divergent thinking, efficiency, emotional enhancement, expectancy, externalizing, extinction, facial expression, familiarity, feature detection, feature-based attention, filtering, focus, framing, generalization, goal, guilt, habit, hallucination, happiness, imagination, incidental learning, intelligence, interference, joint attention, knowledge, language comprehension, language production, lemma, lexical processing, lying, mathematical reasoning, meaning, metacognition, meter, mood, motor sequence learning, navigation, negative priming, overt naming, perceptual identification, phonological processing, positive priming, prosody, prospection, prospective memory, prototype, reinstatement, reward learning, rhythm, rule learning, sadness, semantic memory, shame, social norm processing, source memory, strategy, strength, surprise, sustained attention, task difficulty, task switching, thought, tongue response execution, trait anxiety, uncertainty, utility, verbal fluency, visual search, word generation, working memory retrieval

0.2.2 Image preprocessing

Images were fetched from NeuroVault early 2023 using the Nilearn library [Abraham et al.2014]. We resample all images to the standard MNI152 template and apply the MNI152 brain mask. We tame the effect of outliers by rescaling image values to a standard range, thus losing the statistical scale of the effects. We use the DiFuMo atlas in dimension $d = 1024$ available from Nilearn. As the coefficients of the DiFuMo representation can range to large values, we scale them to a constant to facilitate the convergence of the neural network.

0.2.3 Peak extraction

We generate matching (peaks, image) pairs from the collection 6618 from NeuroVault, without considering the associated labels. Similarly, we apply masking, resampling and DiFuMo compression without the normalization step. We use Nilearn for the peak extraction, which first separates the images into statistically active clusters and identifies local maxima as peak coordinates for each cluster. As these images represent statistical maps, equivalent to p-values of voxel-level tests, we keep peaks with a p-value lower than 10^{-3}, and with a minimal distance between peaks within the same cluster of

8mm. These are standard parameters from peak extraction in brain imaging contexts [Button et al.2013].

0.2.4 Cognitive Atlas association

We present in this paper aggregation of metrics over Cognitive Atlas categories. We find this presentation more easily readable than representing each cognitive concept individually. We parse the mapping of terms upon Cognitive Atlas categories from the Cognitive Atlas website[4]. Additionally, for terms without a mapping, we look for categories assigned to its parents. We present in the table below the mapping between the Cognitive Atlas categories and the cognitive processes used in the paper.

0.2.5 NeuroSynth and NeuroQuery training

Pubget also facilitates the preprocessing of the publications by providing directly the TF-IDF representation of the text. It facilitates the fit of NeuroSynth and NeuroQuery on the set of downloaded publications.

We use those versions to act as our baselines. We make sure that this newly fitted NeuroSynth version matches the original performance on shared terms. We observe similar decoding results, with a slight improvement of the updated version (AUC of 0.663 vs 0.655 for NeuroSynth).

0.3 Peaks2Image training details

0.3.1 Reconstruction

The image reconstruction model is implemented using PyTorch [Paszke et al.2017]. It is a Set Transformer comprising an encoder-decoder architecture applied to each reported peak. The encoding function is composed of 2 induced set attention blocks (ISAB, as defined in the original paper [Lee et al.2019]) with 2 heads and 32 inducing points. The decoder applies a pooling by multihead attention with 2 heads and 1 seed followed by row-wise feed forward layer applied on a set attention block as defined in the original paper [Lee et al.2019]. The output dimension of the model is the chosen DiFuMo dimension of the images, aka 1024. It is trained for 50 epochs using Adam optimizer and a learning rate of 1e-3. The loss is the mean-squared error (MSE) over the DiFuMo components. We select the best set of weights over the validation set, which is composed of 20% of collection 6618.

0.3.2 Decoding

The decoder model is a multi-layer perceptron composed of one hidden layer of 300 units and ReLU activation. It is trained for 200 epochs on the reconstructed neuroscientific publications using the Adam optimizer with a learning rate of 3e-3 on the binary cross-entropy loss. During evaluation, we apply an additional sigmoid operation to the model outputs. We use the Area Under the Receiver Operating Characteristic Curve (ROC AUC) over each label of the testing set to evaluate the decoding performance. Chance level is 0.5. We performed 20 different runs of the experiments with different splits to obtain confidence intervals. AUCs were deemed significant when there 95% confidence intervals was situated above .5.

[4] https://www.cognitiveatlas.org/concepts/categories/all

Main category	Cognitive process
Action	action, effort, left finger response execution, left hand response execution, left toe response execution, motor control, motor planning, response execution, response selection, right finger response execution, right hand response execution, right toe response execution, tongue response execution
Cognitive-affective processes	agency, agreeableness, appetite, association, attitude, balance, behavioral inhibition, belief, binocular rivalry, centration, cognitive effort, competition, conceptual priming, consciousness, crossmodal, cueing, domain specificity, efficiency, externalizing, extinction, feature-based attention, filtering, hallucination, integration, interference, interference control, introspection, loss, loss anticipation, memory trace, metacognition, meter, overt naming, phonological comparison, prospection, punishment processing, reinstatement, response bias, reward anticipation, reward learning, reward valuation, salience, sleep, social norm processing, strength, trait anxiety
Perception	acuity, action perception, audition, auditory coding, auditory masking, auditory scene, auditory sentence recognition, auditory tone detection, auditory tone discrimination, categorical perception, color perception, detection, discrimination, face perception, facial trustworthiness recognition, feature detection, localization, mental imagery, mental rotation, motion detection, navigation, negative priming, object perception, pattern recognition, perception, perceptual identification, positive priming, rhythm, spatial ability, visual form recognition, visual localization, visual object detection, visual object recognition, visual perception, visual recognition, visual sentence recognition
Learning and memory	adaptation, autobiographical memory, body maintenance, concept learning, episodic memory, face maintenance, face recognition, familiarity, forgetting, generalization, habit, incidental learning, instrumental conditioning, instrumental learning, knowledge, learning, memory, memory acquisition, memory retrieval, motor sequence learning, object maintenance, pavlovian conditioning, place maintenance, priming, prospective memory, prototype, reinforcement learning, reward processing, rule learning, source memory, spatial memory, string maintenance, tool maintenance, visual memory
Reasoning and decision making	analogical reasoning, animacy decision, arithmetic processing, categorization, concept, creative thinking, decision, decision certainty, decision making, decision under uncertainty, deductive reasoning, divergent thinking, feature comparison, framing, imagination, inference, intelligence, judgment, logical reasoning, loss aversion, mathematical reasoning, mental arithmetic, numerical comparison, problem solving, reasoning, risk, risk processing, risk seeking, schema, social inference, strategy, thought, uncertainty, utility
Attention	anticipation, attention, attentional blink, attentional state, distraction, fixation, focus, joint attention, search, spatial attention, spatial working memory, sustained attention, visual attention, visual search, visual working memory
Emotion	anxiety, arousal, emotion, emotion perception, emotion recognition, emotion regulation, emotional enhancement, empathy, facial expression, fear, guilt, happiness, mood, negative emotion, pain, pain habituation, sadness, shame, stress, surprise
Executive cognitive control	cognitive control, cognitive load, error detection, goal, inhibition, maintenance, manipulation, monitoring, planning, response conflict, response inhibition, selective control, self monitoring, task switching, updating, working memory, working memory retrieval
Social function	communication, lying, social cognition, theory of mind
Language	conversation, language, language comprehension, language production, lemma, lexical processing, meaning, naming, parsing, phonological processing, prosody, reading, semantic categorization, semantic memory, semantic processing, sentence processing, speech perception, speech processing, story comprehension, verbal fluency, visual word recognition, word generation, word recognition
Motivation	desire, expectancy, task difficulty

Table S1. Mapping of the cognitive processes used in NeuroVault onto the Cognitive Atlas categories. The category of each cognitive term is either directly obtained from Cognitive Atlas categorization or inherited through a parent process category.

0.4 Example of reconstructions

Figure S1 presents three examples of reconstruction with good, fair, and poor accuracy. Interestingly, unlike kernel-based methods, it shows that the reconstruction can have positive and negative regions. We observe that the reconstruction does not consider peak height and can therefore place too much importance on some secondary peaks while missing the dominant pattern in the data. This explains the less accurate reconstruction.

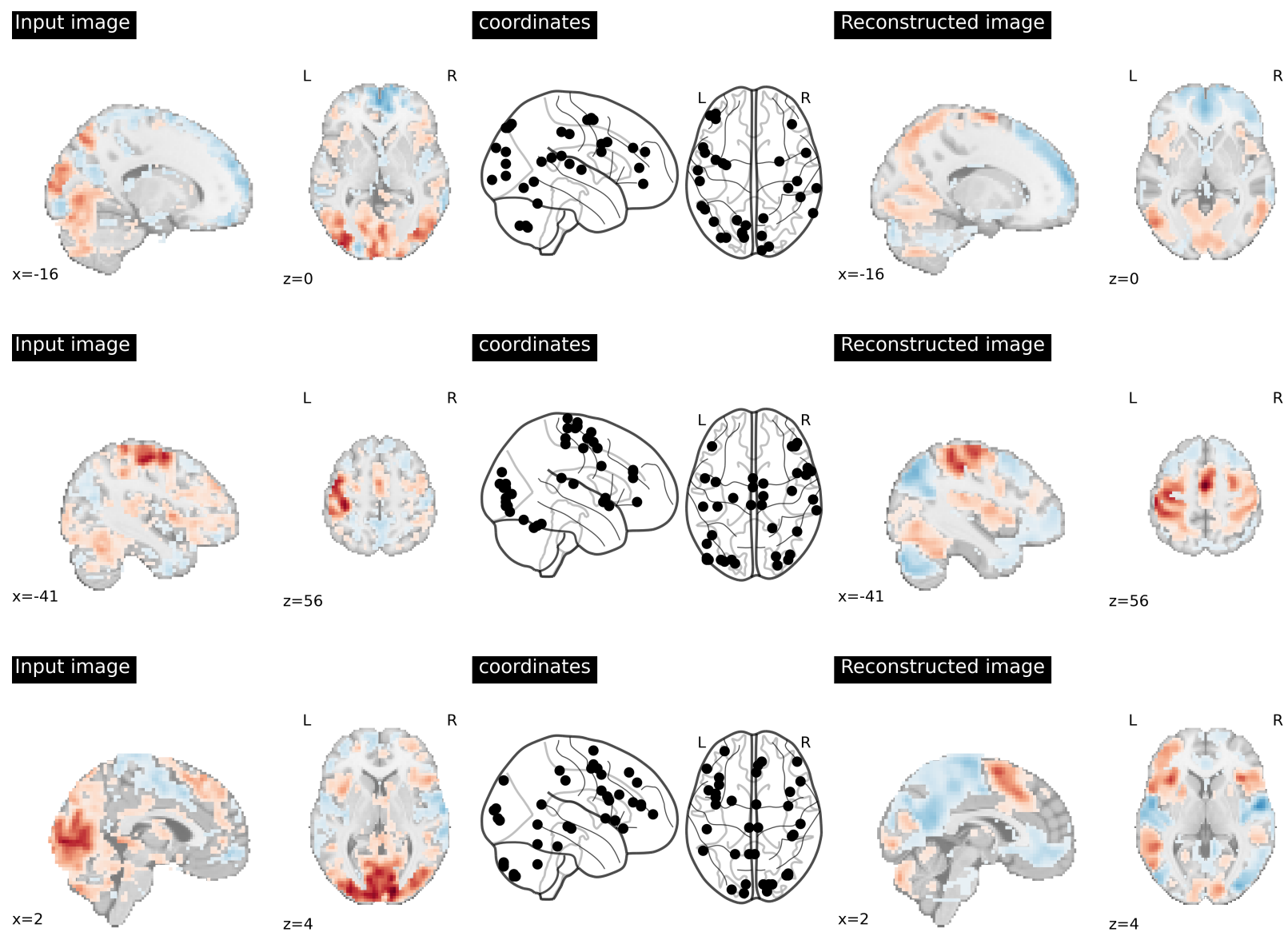

Fig S1. Example of reconstructions withs Peaks2Image. The left column shows the image to be reconstructed. The middle column shows the extracted peak locations. The right column shows the model's output. The top example is an accurate reconstruction (correlation = 0.46); the middle example is a fair reconstruction (correlation = 0.41); and the bottom example is a failed reconstruction (correlation = 0.21). Interestingly, unlike kernel-based methods, the reconstruction can have positive and negative regions. However, we notice that the reconstruction does not take peak height into account and thus may give too much importance to some secondary peaks while missing the dominant pattern in the data.

0.5 Per-sample reconstruction performance

In Fig. S2 we present for each image of Neurovault not used for model training, the correlation of the image reconstruction based on Peaks2Image with the input image. We plot it against the standard kernel-based reconstruction. The average correlation between the input image to be 0.35 for Peaks2Image and 0.27 for the kernel-based estimator.

0.6 Learning from resting-state fMRI

Task-fMRI data are expensive to collect, and require neuroscientist expertise to design and evaluate tasks. They are still available in limited samples. For this reason, learning to reconstruct them from resting-state fMRI could be beneficial to increase statistical power.

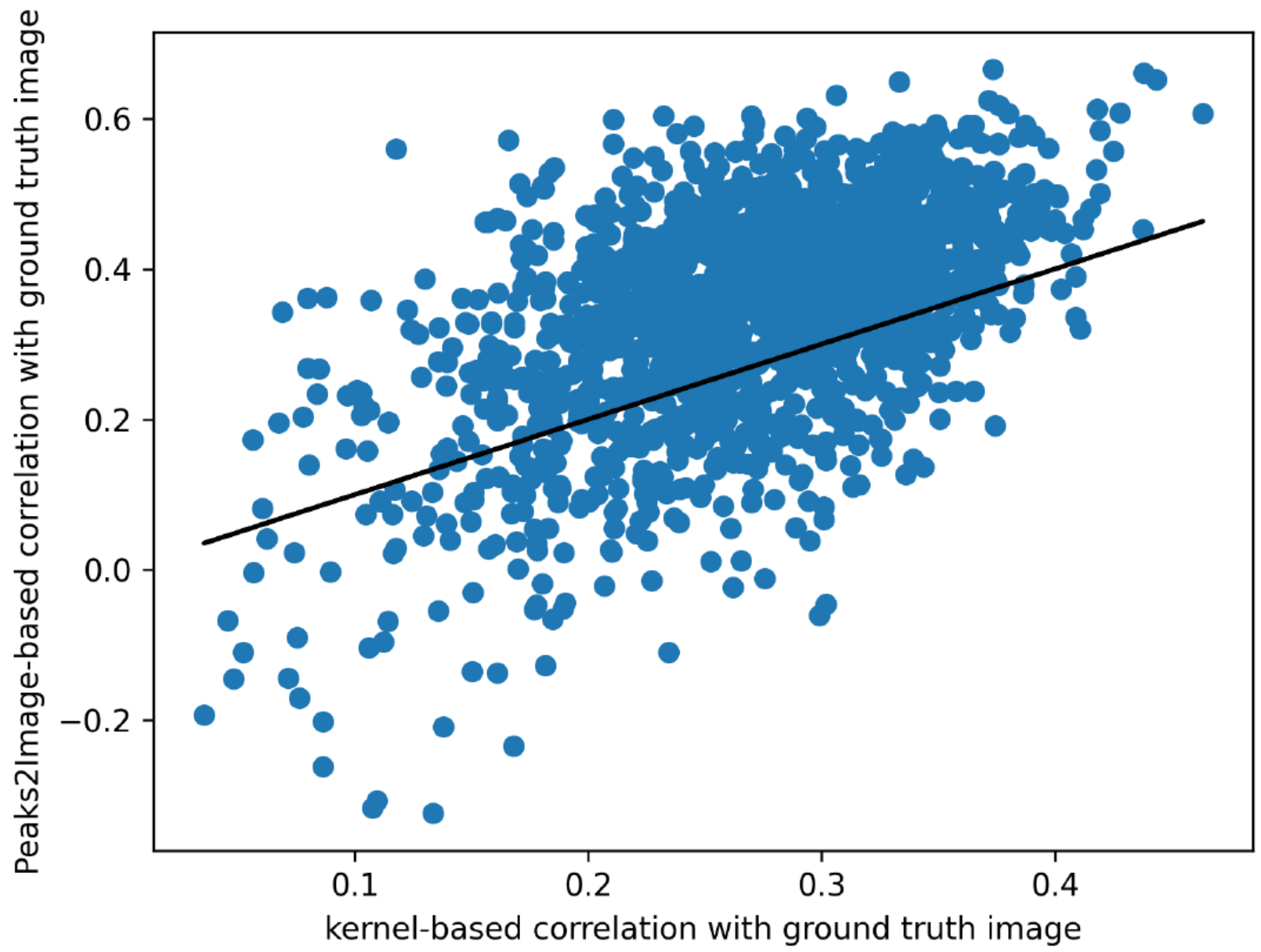

Fig S2. Per-sample reconstruction of performance of Peask2Image. In this plot we present for each image of Neurovault not used for model training, the correlation of the image reconstruction based on Peaks2Image with the input image. We plot it against the standard kernel-based reconstruction. The average correlation between the input image to be 0.35 for Peaks2Image and 0.27 for the kernel-based estimator. Peaks2Image outperforms kernel-based reconstruction by a large margin.

Components found in Independent Component Analysis (ICA) usually represent a wide variety of activations. We aim to generate a large number of those components to learn the reconstruction process, avoiding the use of task-fMRI data. We build a dataset of corresponding peaks and images by applying Canonical ICA (CanICA) [Varoquaux et al.2010] on resting-state data from the Human Connectome Project (HCP) 900 release [Smith et al.2013]. We randomly select sessions from between 10 and 30 subjects of HCP to perform over 2K ICA runs, producing each time between 30 and 60 components through CanICA. By repeating this operation multiple times on different subjects and sessions, we build a set of 21K ICA components. We standardize those components by substracting the mean and reducing them to the unit variance. Those standardized components range to similar values as NeuroVault Z maps. We extracted peak coordinates from these components at different thresholds in {2., 3., 4.} in Z-scale, to vary the number of reported peaks per image. This improved the ability of the model to produce more nuanced statistical images, and better capture co-activation patterns in the peaks.

Among those components, we observe noisy patterns that result from artifacts during acquisition. We remove those components by applying a filter based on their correlation to statistical images. Each component is selected only if it reaches a correlation of 0.3 with at least one statistical image from collection 6618. This selection discards reconstruction patterns that would be related to imaging artefacts commonly obtained in ICA decompositions.

We treat each component as an individual image in the training set. Similarly to statistical images, we normalize those components and extract the peak positions of activated clusters. This change in the training dataset does not affect the architecture and settings of the Peaks2Image model. The predicted output is still a DiFuMo

representation, which can be transformed into a brain image.

Peaks2Image atlas

We plot in fig. S3 the contrast maps over reconstructions obtained from Peaks2Image for several terms. We compute the effect size for each term by first separating publications where the term occurs from publications where it does not.

0.7 Training Peaks2Image with resting-state instead of task-fMRI data

Task-fMRI is expensive to collect and annotate, whereas large amount of resting-state data are easily accessible. We investigate the use of the abundant resting-state data to learn the reconstruction process. We evaluate the ICA-trained model on the same dataset from NeuroVault [Gorgolewski et al.2015], leading to an additional dataset-shift adaptation for this model.

ICA-based Peaks2Image remains on par in terms of Dice but yields low performance on the decoding task. We report the relative AUC performance of the ICA version in appendix. Although many brain patterns are well-captured, the ICA training base is not enough to capture specific patterns necessary for brain decoding. In particular, we observe performance reduction in some specific categories, such as attention or reasoning-related tasks.

We analyzed whether there were some spatial biases in the quality of ICs-based reconstruction. We used the Yeo 17 atlas to compute the per-network relative MSE. Specific networks suffer more during the reconstruction from ICA components: N1 & N2 (visual), and N6 (attentional). We show those differences in reconstruction in fig. S4. This can be put in perspective with the relative strength of ICA in the decoding of different tasks. We plot in fig. S4 the difference in the resulting AUC, as compared with the task model. Overall, the ICA-decoder performs worse, but the attentional tasks show an even larger loss in decoding capabilities.

AUC values per task

The average AUC per task (across folds) is given in Table S2 for 189 tags that were observed in NeuroVault.

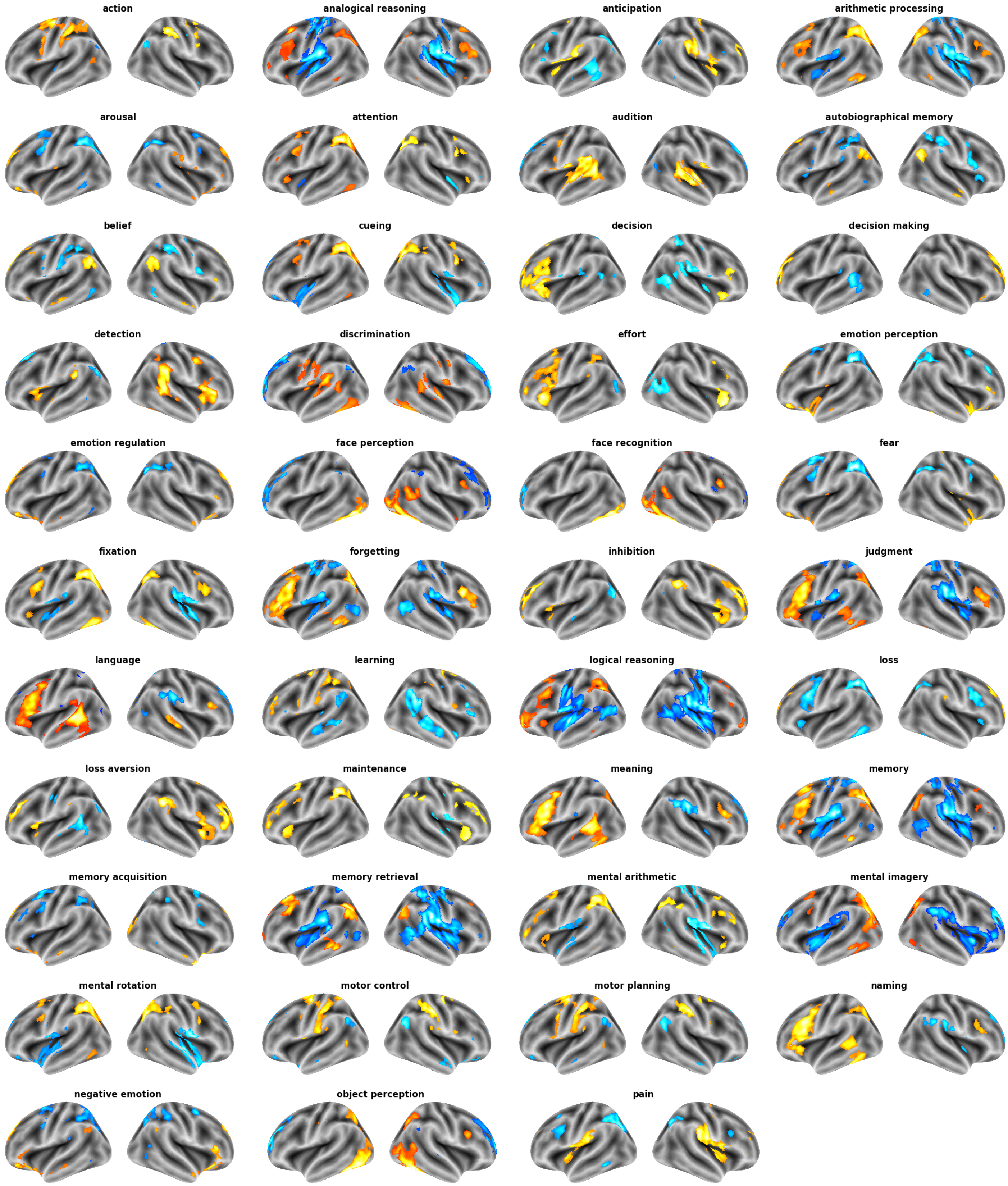
action
analogical reasoning
anticipation
arithmetic processing
arousal
attention
audition
autobiographical memory
belief
cueing
decision
decision making
detection
discrimination
effort
emotion perception
emotion regulation
face perception
face recognition
fear
fixation
forgetting
inhibition
judgment
language
learning
logical reasoning
loss
loss aversion
maintenance
meaning
memory
memory acquisition
memory retrieval
mental arithmetic
mental imagery
mental rotation
motor control
motor planning
naming
negative emotion
object perception
pain

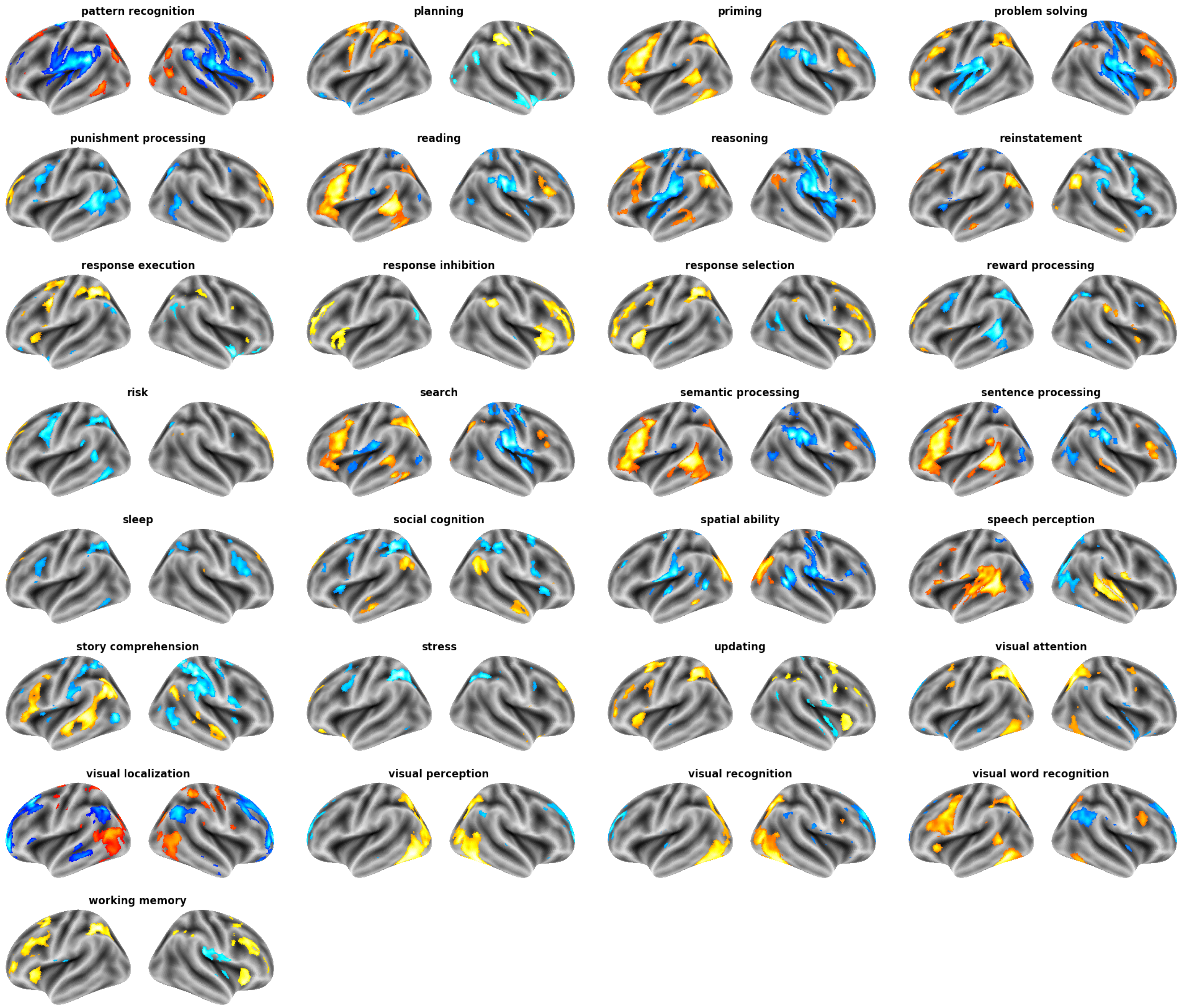

Fig S3. Low-resolution contrast maps for different terms from the vocabulary obtained from the reconstruction of the neuroscientific literature. We project the statistical images onto the surface, and apply a thresholding at the 90th percentile.

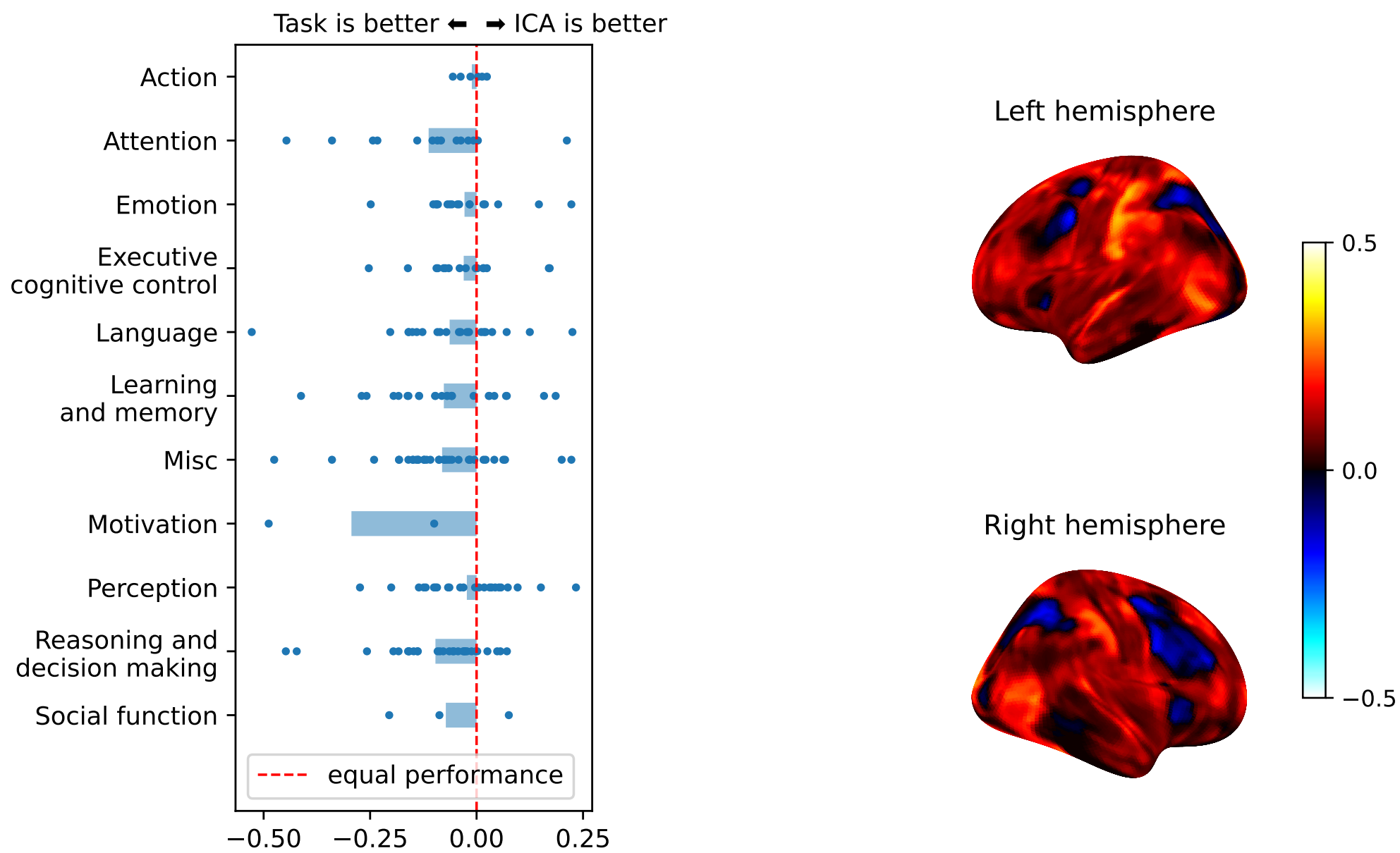

(A) Relative decoding AUC gain of the ICA-based vs task-based Peaks2Image

(B) Relative reconstruction error of the ICA-based vs task-based Peaks2Image

Fig S4. Comparison of ICA-based vs task-based reconstruction: We investigate the effects of learning the reconstruction from resting-state data instead of task-fMRI images. **(A)** Relative AUC between task and ICA-based resting-state representations: for each task, we compare the AUC performance of the models trained on task-reconstructions or the ICs-based reconstructions. The resting-state ICs model performs worse, and tasks associated to brain areas with higher reconstruction errors pose a greater challenge. **(B)** Relative reconstruction error: we show the average AUC difference (bar) and task-specific AUC difference (dot) for ICA vs task-based models. Specific zones suffer more from training on ICA components instead of task data, in particular, the attentional network. This affects the downstream decoding capabilities, where almost all attentional tasks perform worse.

References

Abraham et al.2014. Abraham, A., Pedregosa, F., Eickenberg, M., Gervais, P., Mueller, A., Kossaifi, J., Gramfort, A., Thirion, B., and Varoquaux, G. (2014). Machine learning for neuroimaging with scikit-learn. *Frontiers in neuroinformatics*, 8:14.

Button et al.2013. Button, K. S., Ioannidis, J. P., Mokrysz, C., Nosek, B. A., Flint, J., Robinson, E. S., and Munafò, M. R. (2013). Power failure: why small sample size undermines the reliability of neuroscience. *Nature Reviews Neuroscience*, 14(5):365.

Gorgolewski et al.2015. Gorgolewski, K. J., Varoquaux, G., Rivera, G., Schwarz, Y., Ghosh, S. S., Maumet, C., Sochat, V. V., Nichols, T. E., Poldrack, R. A., Poline, J.-B., et al. (2015). Neurovault. org: a web-based repository for collecting and sharing unthresholded statistical maps of the human brain. *Frontiers in neuroinformatics*, 9:8.

Table S2. Average AUC value per concept

Cognitive Label	AUC	Cognitive Label	AUC	Cognitive Label	AUC
action	0.57	imagination	0.68	reasoning	0.60
acuity	0.88	incidental learning	0.95	reinforcement learning	0.30
adaptation	0.90	inference	0.81	reinstatement	0.77
agency	0.81	inhibition	0.79	response bias	0.72
agreeableness	0.79	instrumental conditioning	0.82	response conflict	0.67
analogical reasoning	0.78	instrumental learning	0.77	response execution	0.54
anticipation	0.69	intelligence	0.96	response inhibition	0.68
anxiety	0.93	interference	0.84	response selection	0.54
arithmetic processing	0.78	interference control	0.83	reward anticipation	0.60
arousal	0.74	introspection	0.57	reward learning	0.81
attention	0.80	joint attention	0.95	reward processing	0.81
attentional blink	0.73	judgment	0.60	reward valuation	0.85
attentional state	0.64	knowledge	0.70	rhythm	0.61
attitude	0.71	language	0.80	risk	0.74
audition	0.83	language production	0.64	risk processing	0.69
auditory scene	0.73	learning	0.73	risk seeking	0.67
autobiographical memory	0.58	lemma	0.83	rule learning	0.63
balance	0.62	lexical processing	0.86	sadness	0.97
behavioral inhibition	0.83	localization	1.00	salience	0.83
belief	0.85	logical reasoning	0.74	schema	0.76
binocular rivalry	0.90	loss	0.83	search	0.77
categorical perception	0.45	loss anticipation	0.67	selective control	0.70
cognitive control	0.79	loss aversion	0.62	self monitoring	0.87
cognitive effort	0.81	lying	0.82	semantic categorization	0.66
cognitive load	0.89	maintenance	0.84	semantic memory	0.64
color perception	0.62	manipulation	0.90	semantic processing	0.83
communication	0.78	mathematical reasoning	0.88	sentence processing	0.82
concept	0.78	meaning	0.83	sleep	0.87
conceptual priming	0.91	memory	0.75	social cognition	0.84
creative thinking	0.78	memory acquisition	0.62	social inference	0.81
cueing	0.36	memory retrieval	0.65	source memory	0.80
decision	0.83	memory trace	0.56	spatial ability	0.57
decision making	0.73	mental arithmetic	0.45	spatial attention	0.64
deductive reasoning	0.78	mental imagery	0.86	spatial memory	0.72
detection	0.94	mental rotation	0.47	spatial working memory	0.68
discrimination	0.62	monitoring	0.84	speech perception	0.86
divergent thinking	0.85	mood	0.91	speech processing	0.73
domain specificity	0.54	motion detection	0.27	story comprehension	0.91
efficiency	0.85	motor control	0.84	strategy	0.96
effort	0.81	motor planning	0.91	strength	0.85
emotion perception	0.32	motor sequence learning	0.83	stress	0.86
emotion recognition	0.34	naming	0.71	surprise	0.82
emotion regulation	0.58	navigation	0.88	sustained attention	0.65
emotional enhancement	0.87	negative emotion	0.32	task difficulty	0.73
empathy	0.86	negative priming	0.74	task switching	0.62
episodic memory	0.73	numerical comparison	0.44	thought	0.90
error detection	0.69	object perception	0.26	trait anxiety	0.87
expectancy	0.92	overt naming	0.55	uncertainty	0.63
extinction	0.94	pain	0.75	updating	0.60
face perception	0.75	parsing	1.00	utility	0.95
face recognition	0.35	pattern recognition	0.21	verbal fluency	0.86
facial expression	0.86	pavlovian conditioning	0.89	visual attention	0.41
fear	0.86	perception	0.83	visual localization	0.63
feature detection	0.81	perceptual identification	0.92	visual memory	0.70
filtering	0.89	phonological processing	0.78	visual object recognition	0.37
fixation	0.74	planning	0.85	visual perception	0.84
focus	0.83	positive priming	0.74	visual recognition	0.55
forgetting	0.89	priming	0.87	visual search	0.66
framing	0.81	problem solving	0.62	visual word recognition	0.74
goal	0.81	prosody	0.96	visual working memory	0.51
guilt	0.94	prospective memory	0.73	word generation	0.62
habit	0.86	prototype	0.71	word recognition	0.76
hallucination	0.93	punishment processing	0.90	working memory	0.77

Lee et al.2019. Lee, J., Lee, Y., Kim, J., Kosiorek, A. R., Choi, S., and Teh, Y. W. (2019). Set Transformer: A Framework for Attention-based Permutation-Invariant Neural Networks. *arXiv:1810.00825 [cs, stat]*. arXiv: 1810.00825.

Menuet et al.2022. Menuet, R., Meudec, R., Dockès, J., Varoquaux, G., and Thirion, B. (2022). Comprehensive decoding mental processes from web repositories of functional brain images. *Sci. Rep.*, 12(1):7050.

Paszke et al.2017. Paszke, A., Gross, S., Chintala, S., Chanan, G., Yang, E., DeVito, Z., Lin, Z., Desmaison, A., Antiga, L., and Lerer, A. (2017). Automatic differentiation in pytorch.

Smith et al.2013. Smith, S. M., Beckmann, C. F., Andersson, J., Auerbach, E. J., Bijsterbosch, J., Douaud, G., Duff, E., Feinberg, D. A., Griffanti, L., Harms, M. P., Kelly, M., Laumann, T., Miller, K. L., Moeller, S., Petersen, S., Power, J., Salimi-Khorshidi, G., Snyder, A. Z., Vu, A. T., Woolrich, M. W., Xu, J., Yacoub, E., Uğurbil, K., Van Essen, D. C., and Glasser, M. F. (2013). Resting-state fMRI in the Human Connectome Project. *Neuroimage*, 80:144–168.

Varoquaux et al.2010. Varoquaux, G., Sadaghiani, S., Pinel, P., Kleinschmidt, A., Poline, J., and Thirion, B. (2010). A group model for stable multi-subject ica on fmri datasets. *NeuroImage*, 51(1):288–299.
